# Body Mass Index, percent body fat, and regional body fat distribution in relation to leptin concentrations in healthy, non-smoking postmenopausal women in a feeding study

**DOI:** 10.1186/1475-2891-6-3

**Published:** 2007-01-17

**Authors:** Somdat Mahabir, David Baer, Laura L Johnson, Mark Roth, William Campbell, Beverly Clevidence, Philip R Taylor

**Affiliations:** 1 Department of Epidemiology, Division of Cancer Prevention and Population Sciences, The University of Texas M.D. Anderson Cancer Center, Houston, TX, USA; 2 US Department of Agriculture, Agricultural Research Service, Beltsville, MD, USA; 3 National Center for Complementary and Alternative Medicine, National Institutes of Health, Bethesda, MD, USA; 4 Nutritional Epidemiology Branch, Division of Cancer Epidemiology and Genetics, National Institutes of Health, Bethesda, MD, USA; 5 Genetic Epidemiology Branch, Division of Cancer Epidemiology and Genetics, National Institutes of Health, Bethesda, MD, USA

## Abstract

**Background:**

The relationship between BMI and leptin has been studied extensively in the past, but previous reports in postmenopausal women have not been conducted under carefully controlled dietary conditions of weight maintenance using precise measures of body fat distribution. The aim of the present study was to examine the association between serum leptin concentration and adiposity as estimated by BMI and dual energy x-ray absorptiometry (DEXA) measures (percent body fat, central and peripheral fat, and lean mass) in postmenopausal women.

**Methods:**

This study was conducted as a cross-sectional analysis within the control segment of a randomized, crossover trial in which postmenopausal women (n = 51) consumed 0 (control), 15 (one drink), and 30 (two drinks) g alcohol (ethanol)/d for 8 weeks as part of a controlled diet. BMIs were determined and DEXA scans were administered to the women during the 0 g alcohol treatment, and a blood sample was collected at baseline and week 8 of each study period for leptin analysis.

**Results and discussion:**

In multivariate analysis, women who were overweight (BMI > 25 to ≤ 30 kg/m^2^) had a 2-fold increase, and obese women (BMI > 30 kg/m^2^) had more than a 3-fold increase in serum leptin concentrations compared to normal weight (BMI ≤25 kg/m^2^) women. When the models for the different measures of adiposity were assessed by multiple R^2^, models which included percent body fat explained the highest proportion (approximately 80%) of the serum leptin variance.

**Conclusion:**

Under carefully controlled dietary conditions, we confirm that higher levels of adiposity were associated with higher concentrations of serum leptin. It appears that percent body fat in postmenopausal women may be the best adiposity-related predictor of serum leptin.

## Background

Natural menopause, a normal aspect of aging, may influence risk of breast cancer [1], the leading contributor to cancer incidence in women in the United States (US). Menopause is associated with a progressive gain in body weight and an increased tendency for central adiposity with advancing age [2]. Thus, the role of adiposity and lean body mass in postmenopausal breast cancer is important because three-fourths of breast cancer cases and deaths occur in women over 50 years of age [3], and general obesity [4] as well as central adiposity [5,6] are risk factors for postmenopausal breast cancer. While it is not completely clear how obesity results in postmenopausal breast carcinogenesis, it has been ascribed to an excess of circulating estrogens resulting from the conversion of androgens into estrogens through aromatization in adipose tissue [7]. Another possibility is that other hormones produced by the adipocytes, such as leptin, may play a critical role in the increased breast cancer risk associated with obesity [8,9]. Circulating leptin concentration is highly correlated with body mass index (BMI) [10,11], and percent body fat [8,9], but less is known about the relations between body fat distribution and lean mass to leptin concentrations in postmenopausal women under carefully controlled dietary conditions of weight maintenance. This is important because both diet and exercise alter serum leptin levels [12-14].

In various *in vitro *models, leptin acts as a growth factor and stimulates cellular proliferation, angiogenesis, motility, and invasion [15-20]. Leptin stimulates the growth of breast cancer cell lines in vitro [18,20] and can induce the expression of proteolytic enzymes which are essential for breast cancer cell invasion [21]. Leptin has also been shown to counteract the anti-tumorigenic activities of anti-estrogens in breast cancer cells [22]. In this study, we assessed the relationship of serum leptin concentrations to adiposity as estimated by BMI, percent body fat, central fat, peripheral fat, and lean mass. The study was conducted under controlled dietary conditions in which the women maintained stable weight. We hypothesized that in postmenopausal women, after controlling for well-known breast cancer risk factors, central fat would be strongly associated with serum leptin concentrations and the association with central body fat would be greater than for BMI.

## Materials and methods

### Study design

This study was part of a randomized, crossover, intervention trial of moderate alcohol supplementation in postmenopausal women (n = 51). Details of the study design and procedures have been published previously [23,24]. Briefly, subjects were assigned to three separate 8-wk study periods during which they consumed a controlled diet and were provided a beverage (orange juice) each day that contained 0, 15, or 30 g alcohol (95% ethanol) in random order. Each subject completed the three *study *periods; each study period was separated by two-five week washout periods. Total and regional adiposity measurements were assessed during the fourth week of the control treatment (0 g alcohol/day). DEXA (Lunar Corp., Model DPX-L, Madison, WI) whole body scans were used for adiposity assessments; measures of BMI were taken on the same day.

### Subjects

Postmenopausal women were recruited by advertisement from the communities surrounding the Beltsville Human Nutrition Research Center, Beltsville, MD. The eligibility criteria were: *(1) *women ≥ 50 y of age, *(2) *postmenopausal (last menses > 12 months before the study started or follicle stimulating hormone > 40,000 mIU/L, natural menopause or hysterectomy with at least one ovary intact), *(3) *not receiving hormone replacement therapy (HRT), *(4) *not taking prescription medications that might interfere with the study, *(5) *willing and able to consume the diet prepared or approved by the Center and no other foods or beverages, and *(6) *without personal or parental history of alcohol abuse. The subjects were evaluated by a physician and determined to be in good health with no signs or symptoms of any disease or endocrine disorders.

This study was approved by the National Cancer Institute's Institutional Review Board and the Committee on Human Research of the Johns Hopkins University Bloomberg School of Hygiene and Public Health. All subjects were fully informed of the study requirements and were required to read and sign a consent form detailing the objectives, risks, and benefits of the study. The subjects were compensated for their participation.

### Diets and feeding

All meals were prepared at the Beltsville Human Nutrition Research Center from typical U.S. foods and served in a seven-day menu cycle. Each day's diet provided 15% energy as protein, 50% energy as carbohydrate, and 35% energy as fat, with a polyunsaturated/monounsaturated/saturated fat ratio of 0.6:1:1. Daily fiber intake was 10 g/1,000 kcal, and daily cholesterol intake was 150 mg/1,000 kcal. Diets provided 100% of the U.S. recommended dietary allowances for vitamins and minerals [25]. The study participants were weighed each weekday by study investigators at the Beltsville facility, and energy intake was adjusted to maintain constant body weight.

### DEXA measurements

Body composition was determined by pencil beam dual energy x-ray absorptiometry (Lunar Corp., Model DPX-L, Madison, WI). Subjects were placed in a supine position with arms and legs close to their body for a whole body scan following the manufacturer's recommended protocol. Whole body and regional lean mass (mass of bone and nonfat soft tissue) and fat mass were determined using the manufacturer's algorithm (software version 1.33).

### Biological sample collection and analysis

During the last week of the control treatment, blood samples for leptin analysis were collected from fasting (> 12 hours) subjects before breakfast (6:30 AM to 9:00 AM) on each of three non-consecutive days in each study period. An equal volume of serum from each day's blood draw was pooled for analysis. Serum was separated and aliquots were frozen at -70°C. The laboratory methods for the serum leptin measurements were described previously [26]. Briefly, circulating leptin concentration was measured in duplicate by radioimmunoassays (RIA) using a commercially available kit (Human Leptin RIA Kit; Linco Research, St. Charles, MO) and quantified using a Cobra Quantum Gamma Counter (Packard Instruments, Downer Grove, IL). Standard reference materials were run as assay controls with each experiment.

### Statistical analysis

Serum leptin concentrations were log transformed using the natural log. All estimates of means and the differences between means were made using the log transformed leptin values. In tables we report means and regression coefficients returned to the original (arithmetic) scale.

Pearson and Spearman correlations between the different DEXA measurements and BMI (kg/m^2 ^calculated from measured weight and height) were determined. Mean serum leptin concentrations for BMI categories were estimated using linear regression models that included a series of indicator variables for three standard BMI categories (normal, ≤ 25 kg/m^2^; overweight, > 25 and ≤ 30 kg/m^2^; or obese, > 30 kg/m^2^). BMI categories were also modeled as ordinal variables with values 0, 1, and 2. Additional models estimated percent changes in serum leptin concentrations per one-unit change in BMI, one-percent change in total body fat (measured as percent body fat), and 1000 g change in central, peripheral, or lean mass modeled as continuous variables. All models included age (continuous), parity (continuous), race (example, African American, yes/no), age of menarche (less than 12, yes/no), and family history of breast cancer (mother or full sister with breast cancer, yes/no). One woman's breast cancer information was missing and she was excluded from models that included breast cancer history. Sensitivity models including her as having or not having a family history of breast cancer did not change analysis conclusions. In a second series of models (Model 2), we added BMI to Model 1 as a covariate The addition of alcohol group assignment order, study period, hysterectomy, duration of menses, years since last menses, nulliparity, and age at first birth (for those with children) did not improve the precision of the estimates and these terms were not included in the final models. There was no evidence of effect modification as assessed by likelihood ratio tests of model fit after the addition of cross-product terms to models that included main effects. Throughout the paper all *P*-values are two-sided nominal (unadjusted) *P*-values. *P*-values for BMI and DEXA measurements were determined using likelihood ratio tests comparing models with the BMI or DEXA term of interest to models without that term. Multiple R^2 ^and F-tests were calculated from the linear regression models. Statistical analyses were performed using S-PLUS (S-PLUS version 6.2 for Windows, Seattle, WA, Insightful Corporation; 2002).

## Results

Fifty-one women successfully completed the entire study and are included in the present analysis. The physical characteristics and reproductive history of the subjects at baseline are provided in Table 1. All the participants were postmenopausal. Their ages ranged from 49.2 years to 78.8 years with a median of 58.2 years. Most (75%) women were white, 22% were black, and 4% were Asian. The median body weight was 73.2 kg (range, 42.1 kg to 117.4 kg); BMI ranged from 17.7 kg/m^2 ^to 42.5 kg/m^2 ^(median 26.9); and total body fat ranged from 7,942 g to 55,756 g (median, 26,808 g), while trunk, leg, and arm fat were of progressively lesser magnitude. Descriptive statistics for serum leptin concentrations are also presented in Table 1.

**Table 1:** Characteristics of the subjects (N = 51) at baseline

Characteristics	Mean	Median	(range)
Age (y)	59.7	58.2	(49.2–78.8)
Height (cm)	163.9	163.1	(152.1–179.7)
Weight (kg)	74.8	73.2	(42.1–117.4)
BMI (kg/m^2^)	27.8	26.9	(17.7–42.5)
Total body fat (g)	29,744	26,808	(7,942–55,756)
Central fat (g)	13,830	13,234	(3,056–26,396)
Peripheral fat (g)	14,299	12,476	(4,163–29,507)
Lean mass (g)	39,608	38,973	(29,651–53,548)
% Body Fat	41.3%	42.5%	(17.8%–55.7%)
Age at menarche (yrs)	12.7	13	(10–16)
Duration of menses (yrs)	33.3	35	(12–45)
Years since last menses	13.1	12	(1–38)
Parity (# of children)	3	2	(0–8)
†Age at first birth (yrs)	23.2	22	(16–36)
Leptin, ng/dL	19.8	17.5	(2.9–78.2)

Characteristics		No	(%)

Race			
White		38	(74.5%)
Black		11	(21.6%)
Asian		2	(3.9%)
*Menopause type*			
Natural		39	(76.5%)
Hysterectomy		12	(23.5%)
*Family history of breast cancer*			
Yes		11	(21.5%)

† Based on n = 43 subjects

Table 2 shows the geometric mean serum leptin concentrations by categories of BMI defined as normal weight, overweight, and obese. As expected, we found a highly significant trend for increased concentrations of leptin with increasing levels of overweight or obesity (*P *< 0.0001). Among obese subjects, serum leptin concentrations were more than three-fold those seen in the normal weight subjects.

**Table 2:** Geometric mean serum hormone concentrations by categories of BMI

BMI CATEGORY					
		*Normal weight*	*Overweight*	*Obese*	
BMI categories		(BMI ≤ 25)	(BMI > 25 to ≤ 30)	(BMI > 30)	*P*-trend^††^
N		20	17	14	
	Model	Mean, 95%, CI	Mean, 95%, CI	Mean, 95%, CI	

Leptin, ng/mL	1^†^	8.59 (6.15–12.02)	16.32 (10.98–24.26)	30.09 (20.61–43.93)	< 0.0001

^† ^Model 1 (n = 51) adjusted for age, race, family history of breast cancer, parity and menarche < 12 years.

Multiple R^2 ^= 0.56

^††^*P*-trend from linear regression models where trend is measured as a continuous value after assigning the normal weight category 0, overweight category 1, and obese category 2. *P*-values for these models where BMI is measured as a non-categorized continuous variable are available in Table 3.

Table 3 shows how much serum leptin concentration changed for a one-unit increase of BMI, a one-percent increase in body fat, and a one-kilogram increase in central fat, peripheral fat, or lean body mass. For model 1 (adjusted for age, race, family history of breast cancer, parity, and menarche < 12 years), we found statistically significant increases in the concentrations of serum leptin for all five measures of adiposity. For example, leptin increased 7.8% (95% CI = 6.6%–9.0%) for each one-percent increase in total body fat. This association held true across different measures of adiposity: leptin increased 10.6% (95% CI = 8.3%–12.8%) for each one-kilogram increase in peripheral fat, 11.9% (95% CI = 9.7%–14.2%) for each one-kilogram in central fat, 8.6% (95% CI = 3.8%–13.7%) for each one-kilogram increase in lean mass, and 10.7% (95% CI = 8.2%–13.2%) for each one-unit increase in BMI. Therefore, a one-unit change for each of these adiposity measures was associated with a substantial change in serum leptin. In Model 2, BMI was added to the covariates in Model 1, and demonstrates that percent body fat, central fat, and peripheral fat all provided additional predictive information about serum leptin concentrations beyond BMI and the other covariates.

**Table 3:** Associations of BMI, percent body fat, central fat (trunk fat), peripheral fat, and lean mass with serum leptin concentrations

	Leptin, ng/mL		
	Δ (95% C.I.)	*P*-value	Multiple R^2^
BMI			
Model 1	10.65 (8.16–13.19)	< 0.0001	0.68
Model 2			
% Fat			
Model 1	7.77 (6.56–8.98)	< 0.0001	0.82
Model 2	7.20 (4.72–9.75)	< 0.0001	0.82
Central fat			
Model 1	11.93 (9.72–14.18)	< 0.0001	0.77
Model 2	11.58 (5.87–17.61)	0.0002	0.77
Peripheral fat			
Model 1	10.55 (8.33–12.81)	< 0.0001	0.72
Model 2	7.98 (2.13–14.16)	0.01	0.73
Lean mass			
Model 1	8.61 (3.76–13.68)	0.001	0.31
Model 2	-0.85 (-4.81–3.25)	0.68	0.68

Δ - Percent change for serum leptin concentration for a one-unit change in each of the adiposity measures (i.e., percent change in leptin concentration per one-unit change in BMI, per 1% increase in total body fat, per 1 kg increase in central and peripheral fat and lean mass).

Model 1: Adjusted for age, race, family history of breast cancer, parity and menarche < 12 years

Model 2: Model 1 + BMI

When multiple R^2 ^was used to assess the strength of the linear associations of the overall models to leptin (Table 3), the percent fat models explained the highest proportion (over 80%) of the variance related to serum leptin concentrations. Overall, the multiple R^2^s shown in Table 3 for all the Model 2s (except lean mass) demonstrate that BMI, percent body fat, central fat and peripheral fat models are all associated with serum leptin concentrations.

## Discussion

The objective of the study was to identify the relationship between well-defined measures of body fat distribution and lean body mass and circulating leptin concentrations in healthy postmenopausal women. Our results showing that postmenopausal women with higher levels of adiposity have higher concentrations of serum leptin confirm the findings from several previous reports which showed positive correlations between adiposity and leptin concentrations [8,9,27-30]. Higher lean mass was also associated with higher serum leptin concentrations, albeit less strongly so than for adiposity measures. To our knowledge, this is the first study to evaluate the associations between DEXA body fat distributions and lean body mass in relation to serum leptin concentrations in healthy postmenopausal women not on HRT under controlled dietary conditions in which energy was balanced to maintain weight of the women. Havel et al. [27] found that postmenopausal women (n = 38) (about half of whom were on HRT) kept on a weight maintenance diet with variable fat content found BMI correlated significantly with plasma leptin levels, and that overweight and obese women compared to normal weight women had significantly higher plasma leptin levels. Thus, our study is in agreement with Havel et al. [27] regarding BMI and percent body fat. However, because we used DEXA scans, our study had a larger array of adiposity measures, and our study also had a larger sample size.

Although our study showed that obese women, compared to normal weight women had a greater than three-fold increase in serum leptin concentrations, we also found evidence that both measures of central and peripheral body fat provided additional predictive information beyond that achieved by BMI and other breast cancer risk factors.

Our adiposity measures are on different scales and it is difficult for example, to compare changes of one percent in body fat versus one kg body fat versus one BMI unit, thus we also used multiple R^2 ^values to summarize these associations. The multiple R^2 ^values from the linear regression models reflect the percent variance explained by each of the adiposity variable models (BMI, percent body fat, central and peripheral fat, and lean mass), all adjusted for the covariates, so that they can be compared. Looking at the multiple R^2 ^values, we found the percent body fat model explains a largest proportion (more than 80%) of the variance associated with serum leptin concentrations, and thus appear to be very strongly associated with serum leptin concentrations. However, all of the R^2 ^values in Table 3, except for lean mass, are very high; thus, we conclude that a simple measure – BMI, which is commonly used in epidemiologic studies – captures a large percent of the variance associated with serum leptin concentrations.

In addition to the previous studies which suffer from inadequate control of diet and energy balance (known confounders of leptin levels) [12-14], in postmenopausal women the influence of HRT on circulating leptin concentrations has not been well defined [3,29,31]. Studies by Di Carlo et al. [32] and Tommaselli et al. [33] show that the use of 17 β-estradiol and other estrogenic compounds such as tibolone and raloxifene prevent postmenopausal body composition changes without significant changes in serum leptin concentrations. Our findings support the view that increased adiposity measured as BMI, percent body fat, central and peripheral fat are all associated with increased leptin exposure in non-smoking postmenopausal women not on HRT. Because the women in our study population were non-smokers and not on hormone treatments, the results may not be generalizable to all postmenopausal women.

Since increased BMI [4] and central fat [34] are associated with increased risk for breast cancer in prospective studies, increased leptin exposure associated with obesity and central adiposity could explain the greater incidence of breast cancer in overweight or obese postmenopausal women. This idea is also supported by the findings from several experimental studies in which leptin stimulated breast carcinogenesis [19,22,35]. However, very few epidemiologic studies have assessed leptin concentrations in relation to breast cancer risk. Two small case-control studies, one in postmenopausal [36] and the other in premenopausal [37] women, and a single prospective study [38] found no association between leptin and breast cancer. Thus, more epidemiological studies are clearly needed to confirm the leptin-breast cancer association in postmenopausal women.

Although our study is limited by its cross-sectional design and modest sample size, the strengths of this study include a homogeneous study population (eg, smokers and women taking HRT were excluded) and measurement stability, which resulted from the use of a carefully controlled diet adjusted to maintain body weight. The DEXA scans employed are considered a reference method for body composition analysis [39]. Although our study was conducted within the control (0 g) segment of the alcohol trial, like all cross-over studies, there may be residual treatment effects and for these reasons the design included a two-five week washout period. We previously reported that alcohol treatment (15–30 g/d) increased serum leptin levels in these postmenopausal women [26].

In conclusion, our study demonstrated that serum leptin concentrations showed striking differences by adiposity levels. Increased exposure to leptin was observed for increased adiposity determined by BMI, percent body fat, central and peripheral fat as well as lean body mass. Although BMI and DEXA adiposity (percent body fat, central fat and peripheral fat) are highly correlated in this cross-sectional study, it remains for further studies to confirm and refine our observations regarding: (i) percent body fat as the best adiposity-related predictor of serum leptin, and (ii) the independent value of central body fat in this prediction even after adjustment for BMI. Because of the well-known arguments for limitations in the use of BMI [40], such as BMI does not discriminate between muscle and fat, these points are particularly important to sort out. For example, for a given BMI, Asians have higher body fat content and higher risk for conditions such as diabetes, high blood pressure and heart disease [41]. In addition, prospective studies of adiposity including BMI, other anthropometric measures, and DEXA will still be needed to fully assess the effects of adiposity on diseases in postmenopausal women, including not only breast cancer but also other major causes of morbidity and mortality such as osteoporosis, diabetes and heart disease.

## Competing interests

The author(s) declare that they have no competing interests.

## References

[B1] Edwards BK, Howe HL, Ries LAG, Thun MJ, Rosenberg HM, Yancik R, Wingo PA, Jemal A, Feigal EG (2002). Annual report to the nation on the status of cancer, 1973-1999, featuring implications of aging on U.S. cancer burden.. Cancer.

[B2] Astrup A (1999). Physical activity and weight gain and fat distribution chnages with menopause: current evidence and research issues.. Med Sci Sports Exerc.

[B3] Kohrt WM, Landt M, Birge SJJ (1996). Serum leptin levels are reduced in response to exercise training, not hormone replacement therapy, in older women.. J Clin Endocrinol Metab.

[B4] Calle EE, Rodriguez C, Walker-Thurmond K, Thun MJ (2003). Overweight, obesity, and mortality from cancer in a prospectively studied cohort of U.S. adults.. N Engl Med.

[B5] Sellers TA, Kushi LH, Potter JD, Kaye SA, Nelson CL, McGovern PG, Folsom AR (1992). Effect of family history, body-fat distribution, and reproductive factors on the risk of postmenopausal breast cancer.. N Engl J Med.

[B6] Sellers TA, Davis J, Cerhan JR, Vierkant RA, Olson JE, Pankratz VS, Potter JD, Folsom AR (2002). Interaction of waist/hip ratio and family history on the risk of hormone receptor-defined breast cancer in a prospective study of postmenopausal breast cancer.. Am J Epidemiol.

[B7] Calle EE, Kaaks R (2004). Overweight, obesity and cancer: Epidemiological evidence and proposed mechanisms.. Nature Reviews.

[B8] Considine RV, Sinha MK, Heiman ML, Kriauciunas A, Stephens TW, Nyce MR, Ohannesian JP, Marco CC, McKee LJ, Bauer TL, al. (1996). Serum immunoreactive-leptin concentrations in normal-weight and obese humans.. N Engl Med.

[B9] Ostlund REJ, Yang JW, Klein S, Gingerich R (1996). Relation between plasma leptin concentration and body fat, gender, diet, age, and metabolic covariates.. J Clin Endocrinol Metab.

[B10] Zhang Y, Proenca R, Maffei M, Barone M, Leopold L, Friedman JM (1994). Positional cloning of the mouse obese gene and its human homologue.. Nature.

[B11] Ahima RS, Osei SY (2004). Leptin signaling. Physiol & Behavior.

[B12] Koutsari C, Karpe F, Humphreys SM, Frayn KN, Hardman AE (2003). Plasma leptin is influenced by diet composition and exercise.. Int J Obes.

[B13] Yannakoulia M, Yiannakouris N, Bluher S, Matalas A, Klimis-Zacas D, Mantzoros CS (2003). Body fat mass and macronutrient intake in relation to circulating soluble leptin receptor, free leptin index, adiponectin, and resistin concentrations in healthy humans.. J Clin Endocrinol Metab.

[B14] Hickey MS, Calsbeek DJ (2001). Plasma leptin and exercise: Recent findings.. Sports Med.

[B15] Sierra-Honigmann MR, Nath AK, Murkami C, Garcia-Cardena G, Papapetropoulos A, Sessa WC, Madge LA, Schechner JS, Schwabb MB, Polverini PJ, Flores-Riveros JR (1998). Biological action of leptin as an angiogenic factor.. Science.

[B16] Harris RBS (2000). Leptin - Much more than a satiety signal. Annu Rev Nutr.

[B17] Baratta M, Grolli S, Tamanini C (2003). Effect of leptin in proliferating and differentiated HC11 mouse mammary cells.. Regul Pept.

[B18] Dieudonne MN, Machinal-Quelin F, Serazin-Leory V, Leneveu MC, Pecquery R, Giudicelli Y (2002). Leptin mediates a proliferative response in human MCF7 breast cancer cells.. Biochem Biophys Res Commun.

[B19] Hu X, Juneja SC, Maihle NJ, Cleary MP (2002). Leptin: a growth factor in normal and malignant breast cells and normal mammary gland development.. J Natl Cancer Inst.

[B20] Laud K, Gourdou I, Pessemesse L, Peyrat JP, Djiane J (2002). Identification of leptin receptors in human breast cancer: functional activity in T47-D breast cancer cells line.. Mol Cell Endocrinol.

[B21] Castellucci M, De Matteis R, Meisser A, Cancello R, Monsurro V, Islami D, Sarzani R, Marzioni D, Cinti S, Bischoff P (2000). Leptin modulates extracellular matrix molecules and metalloproteinases: possible implications for trophoblast invasion.. Mol Hum Reprod.

[B22] Garofalo C, Sisci D, Surmacz E (2004). Leptin interferes with the effects of the antiestrogen ICI 182, 780 in MCF-7 breast cancer cells.. Clin Cancer Res.

[B23] Baer DJ, Judd JT, Clevidence BA, Muesing RA, Cambell WS, Brown ED, Taylor PR (2002). Moderate alcohol consumption lowers risk factors for cardiovascular disease in postmenopausal women fed a controlled diet.. Am J Clin Nutr.

[B24] Dorgan JF, Baer DJ, Albert PS, Judd JT, Brown ED, Corle DK, Cambell WS, Hartman TJ, Tejpar AA, Clevidence BA, Giffen CA, Chandler DW, Stanczyk FZ, Taylor PR (2001). Serum hormones and the alcohol-breast cancer association in postmenopausal women.. J Natl Cancer Inst.

[B25] (1989). National Research Council. Recommended dietary allowances. 10th ed. Washington, DC: National Academy Press..

[B26] Roth MJ, Baer DJ, Albert PS, Castonguay TW, Dorgan JF, Dawsey SM, Brown ED, Hartman TJ, Campbell WS, Giffen CA, Judd JT, Taylor PR (2003). Relationship between serum leptin levels and alcohol consumotion in a controlled feeding and alcohol ingestion study.. J Natl Cancer Inst.

[B27] Havel PJ, Kasim-Karakas S, Mueller W, Johnson PR, Gingerich RL, Stern JS (1996). Relationship of plasma leptin to plasma insulin and adiposity in normal weight and overweight women: effects of dietary fat content and sustained weight loss.. J Clin Endocrinol Metab.

[B28] Kennedy A, Gettys TW, Watson P, Wallace P, Ganaway E, Pan Q, Garvey T (1997). The metabolic significance of leptin in humans: Gender-based differences in relationship to adiposity, insulin sensitivity, and energy expenditure.. J Clin Endocrinol Metab.

[B29] Haffner SM, Mykkanen L, Stern MP (1997). Leptin concentrations in women in the San Antonio heart study: effect of menopausal status and postmenopausal hormone replacement therapy.. Am J Epidemiol.

[B30] Haffner SM, Gingerich RL, Miettinen H, Stern MP (1996). Leptin concentrations in relation to overall adiposity and regional body fat distribution in Mexican Americans.. Int J Obes Relat Metab Disord.

[B31] Gower BA, Nagy TR, Goran MI, Smith A, Kent E (2000). Leptin in postmenopausal women: Influence of hormone therapy, insulin, and fat distribution.. J Clin Endocrinol Metab.

[B32] Di Carlo C, Tommaselli GA, Sammartino A, Bifulco G, Nasti A, Nappi C (2004). Serum leptin levels and body composition in postmenopausal women: effects of hormone therapy.. Menopause.

[B33] Tommaselli GA, Di Carlo C, Sardo AD, Bifulco G, Cirillo D, Guida M, Capasso R, Nappi C (2006). Serum leptin levels and body composition in postmenopausal women treated with tibolone and raloxifene.. Menopause.

[B34] Folsom AR, Kaye SA, Prineas RJ, Potter JD, Gapstur SM, Wallace RB (1990). Increased incidence of carcinoma of the breast associated with abdominal adiposity in postmenopausal women.. Am J Epidemiol.

[B35] Yin N, Wang D, Zhang H, Yi X, Sun XD, Shi B, Wu H, Wu G, Wang X, Shang Y (2004). Molecular mechanisms involved in the growth stimulation of breast cancer cells by leptin.. Cancer Res.

[B36] Mantzoros CS, Bolhke K, Moschos S, Cramer DW (1999). Leptin in relation to carcinoma in situ of the breast: A study of pre-menopausal women.. Int J Cancer.

[B37] Petridou E, Padadiamantis Y, Markopoulos C, Spanos E, Dessypris N, Trichopoulos D (2000). Leptin and insulin growth factor I in relation to breast cancer (Greece).. Cancer Causes and Control.

[B38] Stattin P, Soderberg S, Biessy C, Lenner P, Hallmans G, Kaaks R, Olsson T (2004). Plasma leptin and breast cancer risk: a prospective study in northern Sweden.. Breast Cancer Res Treat.

[B39] Panotopoulos G, Jean CR, Bernard GG, Arnaud B (2001). Dual x-ray absorptiometry, bioelectrical impedance, and near infrared interactance in obese women.. Med Sci Sports Exerc.

[B40] Prentice AM, Jebb SA (2001). Beyond body mass index.. Obesity Rev.

[B41] Mandavilli A, Cyranoski D (2004). Asia's big problem (News Feature). Nature Med.

